# Interaction with Patients as a Budding Doctor: An Experience

**DOI:** 10.31729/jnma.7555

**Published:** 2022-08-31

**Authors:** Preeti Yadav

**Affiliations:** 1 Nepalgunj Medical College and Teaching Hospital, Kohalpur, Banke, Nepal

**Keywords:** *communication*, *empathy*, *medical students*, *healthcare quality*

## Abstract

Every medical student amidst COVID-19 missed out on an integral part of medical education which is interaction with patients as all the physical classes were postponed for months. Effective interaction with patients is the heart of medicine and it provides a synergistic effect on the delivery of high-quality care to the patient. However many medical students underestimate the art of communication. This article highlights the importance of basic communication skills; also encourages reflecting upon the whole experience and extracting the learning to apply to any further interactions.

## INTRODUCTION

Communication is the cornerstone of the relationship with the patient in all medical settings with the main aims of creating a good interpersonal relationship, exchanging information, and making treatment-related decisions.^1^

Being medical personnel requires one to get your mind around the patient's situation. There might be a whole tsunami going on in your life, or it might just be a day in your hospital but for the patient, it's a onetime experience full of suffering. Understanding this and making the patient feel at ease will pave the path for quality care for the betterment of the patient.

## MY EXPERIENCE

From taking history to performing the examinations that we're aware of, a level of empathy must be established. In the final year of the university examination, an old lady from a rural district was assigned to me. It was a great start but as time went by our endless questions pushed her to be at unease as such questions may not have been anticipated; and being in a certain stress zone, helplessness was felt due to the limited amount of time and the constant pressure of the university examination. Situations such as travelling out of the home and dealing with illness are constantly dealt with by a patient. A patient under such distress and fear was being interviewed with the examination in due. Despite the time constraints, the only way out was to get the patient at ease. The uneasiness had to be sorted out first. An informal introduction session was begun. The conversation was led by the patient towards other things in her life not limited to her health such as her old days, her place and her family and she was told about mine. Further in the conversation, the procedures of history taking and examination were explained and its importance for both parties. It was a surprise that everything started to fall right into place and the history and examinations went on easier. Great enthusiasm was shown while explaining everything to the patient; she was willing to be examined compared to before when she was reserved.

Back in my days of clinical postings, when tasked with extracting history and examining a patient with an inguinal hernia. The patient was hounded, very much to my distaste, by my colleagues. The unwillingness to provide any information regarding his disease or consent to examination was visible in his behaviour. He was irritated, angry, and shouting at us. Anybody could feel it in the air that the patient was annoyed and rightly so after having his personal space invaded. Our endeavour of extracting history was going nowhere. For a traditional Nepalese elderly to disrobe in front of unknown people let alone in front of females would be difficult and understandably so but, some young minds weren't getting any hint. Thus, to successfully extract the history and perform an examination our approach had to be revisited and a rapport had to be established before anything.

Together a step was taken to introduce ourselves, where life as a medical student was discussed and also about the patient, his family and his profession. Gradually, the hostile air around him settled. A lead was taken by him in the conversation about his family, his life stories, and the reason for hospitalization. It was as if he was an open book and a few sentences had to be just picked for documentation. He even agreed to be examined behind the curtain to maintain his privacy. In the end, he mentioned he did not like the way he was asked questions initially as if it were a police inquiry as the initial questions were quite straightforward regarding the history. He suggested that we need to consider the situation the patients are in and approach them gently. The good thing that happened that day was the take-home messages we got from the patient himself; to approach gently, ask if it's all right if we ask questions or if it's all right to examine. A lesson was learnt on how important it is to feel secure and to make room for some trust before letting anyone deal with his/her illness. The bad part was that we were all final-year medical students and we were on the verge of learning how to make the patient comfortable which instead should have been the first thing that we all should have known about.

Some patients are found to be extremely friendly. For instance, we came across a patient who was extremely humble and described whatever she was asked and all with a smile. This could happen when the interaction is started with a proper greeting to the patient and the patient party along with the kindness that we have to offer during the conversation. The responsibility to provide the same level of effort to patients like her should be fulfilled. Inspiration can be found anywhere and following the history and examination session with her, we felt moved by how she faced the whole situation. Had not been for that experience we'd have missed out on a lot.

As an intern Doctor, there is an opportunity to analyze the situation from both sides. During Medical school, there was a more theoretical portion of the syllabus and the exposure to patients was low which was also influenced by COVID. Anyone in this profession is expected to be humane, compassionate, kind, understanding, and sympathetic.

## WHY IS IT IMPORTANT FOR US AND THE PATIENTS?

Communication is the most important component of good medical practice and of patient-centred care, which has been defined as moral philosophy, which values considering patients' needs, wants and perspectives offers patients opportunities to provide input into and participate in their care and enhance partnership and understanding in the doctor-patient relationship.^2^ Communication skills that we build all along the journey is what shapes us to be the better compatible doctor that patients are comfortable with sharing their deepest problematic experiences, and this marks the first step toward the treatment.

Aside from theory, the hours that we invest in our clinical classes talking to the patient, taking their history, clinical examination, and looking at their charts and management also prepare us in some way for this transition into the practical world.^3^ We need to understand how important it is to practice good communication and to understand how sensitive we need to be.

Empathy, being a multi-construct concept, includes, but is not limited to, physician-patient interactions, interprofessional practice, self-compassion, empathy for others' pain, professional identity formation, stress awareness, and self-reflection and communication. Empathetic communication in patient-physicians interactions fosters information exchange and the impact of understanding and adherence to management plans, which lead to an early return to work, pain relief, mood elevation, and improved functional status of patients.^4^

The patient being caught up in that unhealthy situation will seem less attacking if they know that the people he/she's with at the hospital cares. This will uplift the mental status and thus impose a positive effect on the betterment of the patient. To build that level of comfort and way is in our hands, and even that little piece of information can help leap across hurdles in terms of diagnosis sometimes. So, now we can imagine how important it is to have a real conversation with our patients. With this learning, I'm sure the patient will feel more cared for and it carries utmost importance in our profession. Apart from improving patients' feelings, I'm sure this learning will help my patient's outcomes.

"The patient will never care how much you know until they know how much you care".^5^

It is extremely important to be truthful, humble, and unassertive to be friendly and approachable while speaking to patients of all ages in addition to politeness and revealing our position as an intern or medical students. A patient may be in distress due to critical illness or psychological stress. When approaching such patients, our role as medical students or medical interns is different from that of senior Doctors. It stands without reason that we have to command a certain level of knowledge and expertise to be able to identify worsening critical health status which only comes with diligence and enough clinical exposure. So, our role would be to identify the situation with the patient immediately if it is critical illness or psychological stress followed by informing the senior doctor and following the instructions. Critical illnesses may demand immediate medical intervention such as CPR, intubation, or medication and psychological ones may need counselling.

## WHAT CAN BE DONE?

There was a huge setback when the physical classes got cancelled amidst COVID-19; as there were no interactions with the patients meanwhile it was important to catchup on every other patient to fill the missed out part and reflect upon the whole experience, and extract the learning for further interactions.

Supportive student-doctor relationships, studentcentred education, and guidance that address the needs of the doctor-as-person are central to the development of patient-centredness. Medical education requires patient-centred, self-caring, and self-aware role models.^6^ Thus, equal attention and curiosity must be generated to get a step closer to disseminating quality care and organized medical programs.

Therefore, It cannot be emphasized enough how important it is to have our way with words. In addition to medical knowledge, the speech and knowledge of the patient are also important. The perseverance and effort to know about patients' progress in the medical ward also help in making decisions should anything distressful happens to them. Being able to interpret ECG, learning ACLS protocols, knowledge of emergency drugs, their doses, and mode of administration, and knowledge of diseases of a medical emergency are a few which help polish the never-ending pursuit of knowledge.
